# Upregulation of miR-370 and miR-543 is associated with reduced expression of heat shock protein 40 in spinocerebellar ataxia type 3

**DOI:** 10.1371/journal.pone.0201794

**Published:** 2018-08-07

**Authors:** Bernd O. Evert, Rohit Nalavade, Johannes Jungverdorben, Frank Matthes, Stephanie Weber, Ashish Rajput, Stefan Bonn, Oliver Brüstle, Michael Peitz, Sybille Krauß

**Affiliations:** 1 Department of Neurology, University of Bonn, Bonn, Germany; 2 German Center for Neurodegenerative Diseases (DZNE), Regulatory RNA-protein interactions in neurodegenerative diseases, Bonn, Germany; 3 Institute of Reconstructive Neurobiology, University of Bonn LIFE & BRAIN Center and LIFE & BRAIN GmbH, Bonn, Germany; 4 Institute of Medical Systems Biology, Center for Molecular Neurobiology (ZMNH), University Medical Center Hamburg-Eppendorf (UKE), Hamburg, Germany; 5 German Center for Neurodegenerative Diseases (DZNE), Tübingen, Germany; Universitat des Saarlandes, GERMANY

## Abstract

Molecular chaperones are important regulators of protein folding and proteasomal removal of misfolded proteins. In spinocerebellar ataxia type 3 (SCA3), the co-chaperone DnaJ homology subfamily B member 1 (DNAJB1 or heat shock protein 40) is recruited to protein aggregates formed by the disease-causing mutant polyglutamine (polyQ) protein ataxin-3 (ATXN3). Over-expression of DNAJB1 reduces polyQ protein toxicity. Here, we identified two miRNAs, miR-370 and miR-543, that function in posttranscriptional regulation of DNAJB1 expression. MiRNAs are small endogenously produced RNAs controlling mRNA stability and play a role in polyQ disease pathogenesis. In human neuronal cultures derived from SCA3 patient-specific induced pluripotent stem cell (iPSC) lines, miR-370 and miR-543 levels are upregulated, while DNAJB1 expression is concurrently reduced. These findings suggest that downregulation of DNAJB1 by these two miRNAs is an early event that could contribute to SCA3 pathogenesis. Inhibition of these two miRNAs in turn could stabilize DNAJB1 and thereby be beneficial in SCA3 disease.

## Introduction

Chaperones are responsible for modulating protein folding. In several inherited neurodegenerative diseases protein misfolding is a common pathogenic feature [1]. For example the group of polyglutamine (polyQ) diseases is characterized by protein misfolding and aggregation of proteins in which a polyQ tract is abnormally expanded [2]. Spinocerebellar ataxia type 3 (SCA3) is the most common spinocerebellar ataxia and belongs to the group of polyQ diseases [3]. The disease-underlying mutation is an abnormally expanded stretch of the trinucleotide CAG in the *Ataxin 3* (*ATXN3*) gene, which is translated into a polyQ stretch in the ATXN3 protein. At early disease stages mutant ATXN3 induces cell stress pathways, resulting in increased expression of molecular chaperones in an attempt to suppress misfolding of aggregated mutant ATXN3 [4–7]. At later stages, expression of molecular chaperones show a progressive decline impairing the cellular protein quality control [5, 6, 8].

DnaJ homology subfamily B member 1 (DNAJB1), also known as heat shock protein 40 (HSP40), is a co-chaperone that interacts with heat shock protein 70 (HSP70) [1]. Interaction with DNAJB1 stimulates HSP70 ATPase activity and the association between HSC70 and the heat shock protein 70 interacting protein (HIP). DNAJB1 localizes to mutant ATXN3 aggregates [9, 10], while over-expression of DNAJB1 reduces polyQ protein toxicity [11]. This suggests that one pathogenic mechanism in SCA3 involves sequestration of DNAJB1 by mutant ATXN3, which in turn promotes toxicity.

miRNAs are small endogenously produced non-coding RNAs that regulate protein expression from their target mRNAs. They undergo several intracellular cleavage steps to produce short (~20 nucleotide long) RNAs that can be loaded on the RNA induced silencing complex (RISC), where the miRNA acts as a guide to its target mRNA transcripts (reviewed in [12]). The miRNA binding sites are often located in the 3’UTR region of their target mRNA [13]. Nucleotides 2–6 of the miRNA, the so-called “seed region”, are responsible for target recognition: miRNAs that perfectly match their target mRNA sequences induce mRNA degradation, while imperfect matching results in translational repression of the target mRNA [14, 15].

Here, we identified two miRNAs, miR-370 and miR-543, which efficiently target and downregulate the protein expression of DNAJB1. In a SCA3 patient-derived iPS cell model, we found that these two miRNAs are significantly upregulated, while DNAJB1 is concurrently reduced, suggesting that both miRNAs act cooperatively in downregulating DNAJB1 expression. In addition, our findings indicate that deregulated expression of DNAJB1 and its targeting miRNAs already occurs at early stages of SCA3 disease ahead of inclusion formation in human iPSC-derived neurons. Thus, altered expression of miR-370 and miR-543 may contribute to SCA3 pathogenesis by targeting DNAJB1.

## Material and methods

### Prediction of miRNA binding sites

miRNA binding sites in the DNAJB1 mRNA (NM_006145) were predicted using TargetScanHuman 6.2 (http://www.targetscan.org).

### miRNA mimics transfections and western blot

Chemically synthesized, double-stranded RNAs mimicking mature endogenous miRNAs were used for transfection into HeLa cells (ATCC). 100,000 cells per well of a 12-well plate were seeded one day prior transfection. 30 picomoles per well of miRNA mimics (Syn-hsa-miR-370-5p miScript miRNA Mimic and Syn-hsa-miR-543 miScript miRNA Mimic, both from Qiagen) were transfected using lipofectamine 2000 (Invitrogen) according to the manufacturer’s instructions. 48 hours after transfection cells were harvested and lysed in SDS PAGE Sample buffer 2x (25mM EDTA, 100mM Tris, 20% Glycerol, 4% SDS, 2% 2-Mercaptoethanol, 0.004% Bromophenol blue). Mouse brain lysates were kindly provided by Dr. C. Cemal (Imperial College of Science, Technology and Medicine, London, UK) and dissolved in SDS PAGE Sample buffer 2x. Samples were boiled for 5 min at 95°C and proteins were analyzed on 10% SDS gels and blotted onto PVDF-membranes (Roche). Blots were blocked in milk and incubated with the following antibodies: ATXN3 (1:1000; 986, [16]), DNAJB1, (1:1000; Cell Signalling 2118L), GAPDH (1:5000; Cell Signalling, 2118L).

The resulting bands were quantified using AIDA software. Statistical analyses were performed using one-way ANOVA with post hoc Dunnett’s test to accommodate for multiple comparisons or Student’s TTest for two-group comparisons, as appropriate.

### Luciferase reporter assays

The sequence of the 3’ UTR of DNAJB1 containing the miRNA binding sites of interest was PCR amplified (primer sequences: CCGCGGCTCGAGATAGCTATCTGAGCTCC and TATCATGCGGCCGCGAGGTTTAGCATCAGTC) and cloned downstream of the Renilla luciferase gene in the psiCHECK-2 vector using the restriction enzymes *Xho1* and *Not1*. The vector also contains firefly luciferase, which is used for signal normalization.

This construct was used for site directed mutagenesis of miRNA binding sites in 3’UTR sequences. Complementary forward and reverse primers were designed to include the intended base pair substitution mutations in the middle of the primer sequence with flanking regions of unmodified sequence on both sides. Primer sequences were: miR370-1-forward CATCAGGTGGTGGGAACAGCGTGAAAAGGCATTCCAGTC, miRNA370-1-reverse GACTGGAATGCCTTTTCACGCTGTTCCCACCACCTGATG, miR370-2-forward CAATACCTCTCGTTCCAGCGTGACCAAGGGAGCCAGC, miR370-2-reverse GCTGGCTCCCTTGGTCACGCTGGAACGAGAGGTATTG, miR543-forward GGCTTTCGTACTGCTGAATCATTTCCAGAGCATATAT, miR543-reverse ATATATGCTCTGGAAATGATTCAGCAGTACGAAAGCC.

The psiCHECK-2 vectors with the 3’UTR of DNAJB1 carrying the mutations in the insert were PCR amplified, 1 μL of *DpnI* restriction enzyme was added to the PCR product and the product was incubated at 37°C for 2 hours to digest the non-mutated vector. All constructs were verified by Sanger sequencing.

100,000 HeLa cells/well of a 12-well plate were seeded 24 hours prior to transfection. Cells were transfected with psiCHECK-2 constructs using Lipofectamine 2000 according to the manufacturer’s instructions. Cells were washed with PBS and lysed in passive lysis buffer (Promega). Lysates were diluted to attain a concentration of 1 μg/μL. 10 μg of the protein lysate was used per reaction. Each sample was analysed in triplicates, for both the renilla and firefly luciferase measurements. 40 μL substrates for firefly (10 mL Solution A (120 mM Tricine pH 7.8, 15 mM MgSO_4_, 3 mM ATP, 5 mM DTT, 0.27 mM Coenzyme A) + 0.2 mL Solution B (100 mM D-Luciferin) + 29.8 mL H_2_O) or renilla (0.04 mM Coelenterazine) luciferase were added per sample. The luciferase assays were conducted using an Envision plate reader (Perkin Elmer).

### Cultivation and neuronal differentiation of iPSCs

We have used a panel of 3 patient cell lines (patient 1: male, repeat length: 74/21, age at time of biopsy: 40, age of onset 30; patient 2: male, repeat length: 74/22, age at time of biopsy: 38, age of onset 31; patient 3: female, repeat length: 73/27, age at time of biopsy: 42, age of onset 28) and two control cell lines (control 1: female, age at time of biopsy: 24; control 2: male, age at time of biopsy: 68, non-affected father of patient 1). The patient cell lines and control 2 as well as culture conditions have been described in detail previously [17]. Control 1 was purchased at Ebsic (https://cells.ebisc.org/UKBi005-A).

### miRNA expression profiling in iPSCs

Total RNA extraction including small RNAs from iPSC derived neurons was conducted using the miRVana miRNA isolation kit according to the manufacturer’s instructions. The miRNA expression profiling was done by RNA-sequencing on an Illumina HiSeq2000TM with libraries prepared according to the Illumina TruSeq small RNA protocol. The FASTQ files generated for the gene expression profiling were used for analysis by the CLC Workbench. The gene expression profile data were analysed using the CLC genomics workbench. RNA-seq files for the gene expression profile were imported to the CLC server. The sequences were trimmed using default parameters to remove low quality sequences, ambiguous nucleotides and sequences below specific, defined length. The sequences were then assembled, i.e. they were aligned by comparing to reference sequences from GenBank, in order to make contiguous sequences. Default parameters were used for sequence assembly. The control and SCA3 patient samples were grouped into ‘control’ and ‘patient’ groups respectively. An experiment was then set up to analyse the differential expression of genes between the control and patient groups. Results were displayed including parameters such as mean expression values in the groups, fold changes in gene expression between the two groups with the p-values corrected for False Discovery Rate (FDR). Small RNA data was anlaysed with the web application Oasis using standard parameters [18]. In brief, differential gene expression was analyzed using DESeq using standard settings for normalization and differential expression estimation. Genes with a Benjamini & Hochberg FDR below 0.05 were considered to be differentially expressed.

### Real-time PCR

Total RNA was isolated using the RNeasy Mini Kit (Qiagen) according to the manufacturer’s instructions. miRNAs were isolated using miRVana miRNA isolation kit. cDNA was synthesized using the TaqMan reverse transcription reagents kit (Applied Biosystems) and real-time PCR was carried out using the SYBRGreen PCR master mix (Applied Biosystems). Primers sequences were: DNAJB1_forward GCAGTCTTGATTCCCAGACC, DNAJB1_reverse GCTGGAACGAGAGGTATTGC, GAPDH_forward CCACCCATGGCAAATTCC, GAPDH_reverse TGGGATTTCCATTGATGACAAG.

## Results

### MiR-370 and miR-543 target the 3’UTR of DNAJB1 at specific binding sites

**Fig 1:**
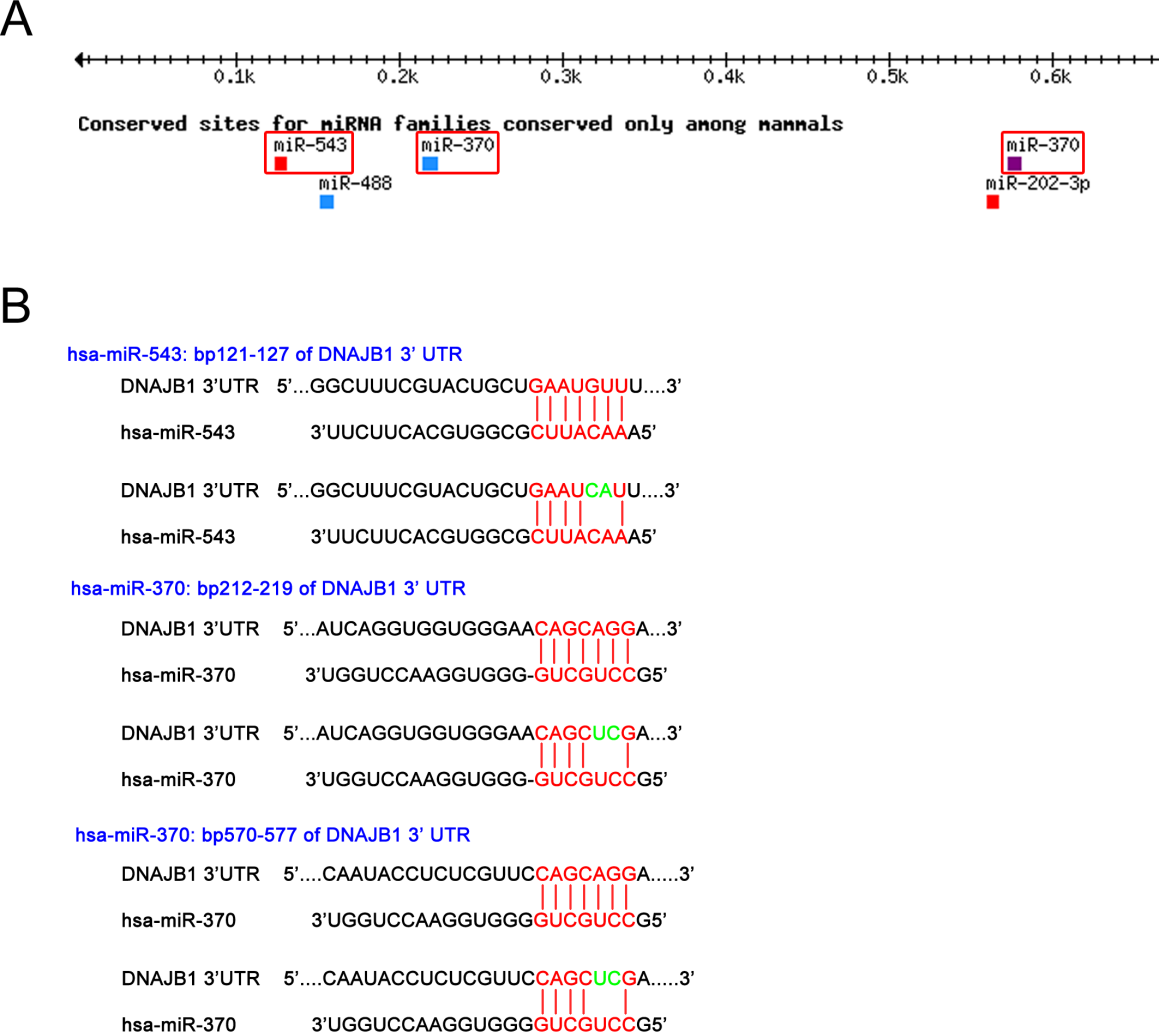
MiRNA binding sites in the 3’-UTR of DNAJB1. (A) Targetscan prediction of miRNAs targeting within the first 700bp of the DNAJB1 3’-UTR. (B) Schematic drawing of the mutations that were inserted into the DNAJB1 3’-UTR luciferase reporter constructs to mutate the seed region of the specific miRNAs.

To identify miRNAs that regulate expression of DNAJB1, we performed a target scan prediction. Two miRNAs, miR-370 and miR-543, were predicted to target DNAJB1 with a percentile score above 80%. miR-370 has two binding sites, both with 8 nucleotide complementarity, which is predicted to have a strong binding ability, whereas miR-543 has a single binding site with 8 nucleotide complementarity (Fig 1A). To test the ability of these two miRNAs to target DNAJB1 mRNA at their specific binding sites we subcloned 1170 bp of the DNAJB1 3’UTR into a luciferase reporter vector (pSICHECK2). The resulting construct encodes the 3’UTR of DNAJB1 fused to Renilla luciferase and a second reporter gene, firefly luciferase, to allow normalization of Renilla luciferase expression. MiRNAs targeting the 3’UTR of DNAJB1 mRNA should then lead to a decrease in Renilla luciferase activity. The DNAJB1-3’UTR construct was further used to mutate the binding site of miR-543 and either or both of the binding sites of miR-370 (Fig 1B).

**Fig 2:**
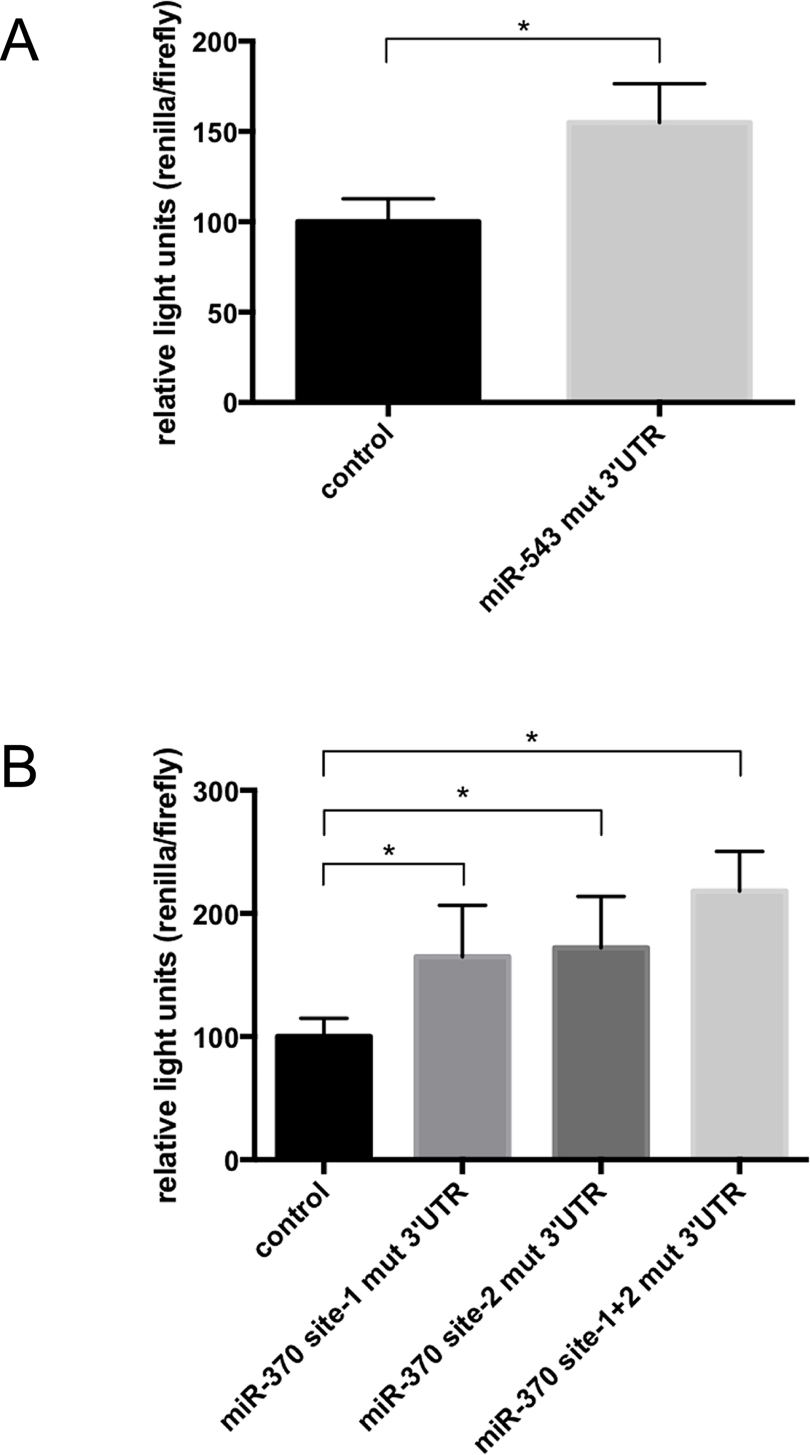
miRNA-370 and miRNA-543 target the 3’UTR of DNAJB1 at specific sites. Luciferase reporter assays of constructs in which the 3’ UTR of DNAJB1 is linked to Renilla luciferase were performed. Firefly was present on the same plasmids and was used for normalization. (A) The binding site of miR-543 on the 3’ UTR of DNAJB1 was mutated. (B) The two binding sites of miRNA-370 on the 3’ UTR of DNAJB1 mRNA were mutated. Columns represent mean values +/- SD of n = 27 samples, p < 0.0001.

These constructs were transfected into Hela cells and luciferase activities were measured. Compared to the wildtype 3'UTR-DNAJB1 reporter construct, a significant increase in the ratios of Renilla/firefly activities was observed for constructs with mutations in the miR-543 binding site (Fig 2A) as well as constructs with one or both miR-370 binding sites mutated (Fig 2B). This finding suggests that endogenously expressed miR-370 and miR-543 are able to target the wildtype 3’ UTR of DNAJB1 at their respective binding sites but are unable to target the mRNA containing the mutated binding sites in the 3’ UTR of DNAJB1.

### MiR-370 and miR-543 target endogenous DNAJB1

**Fig 3:**
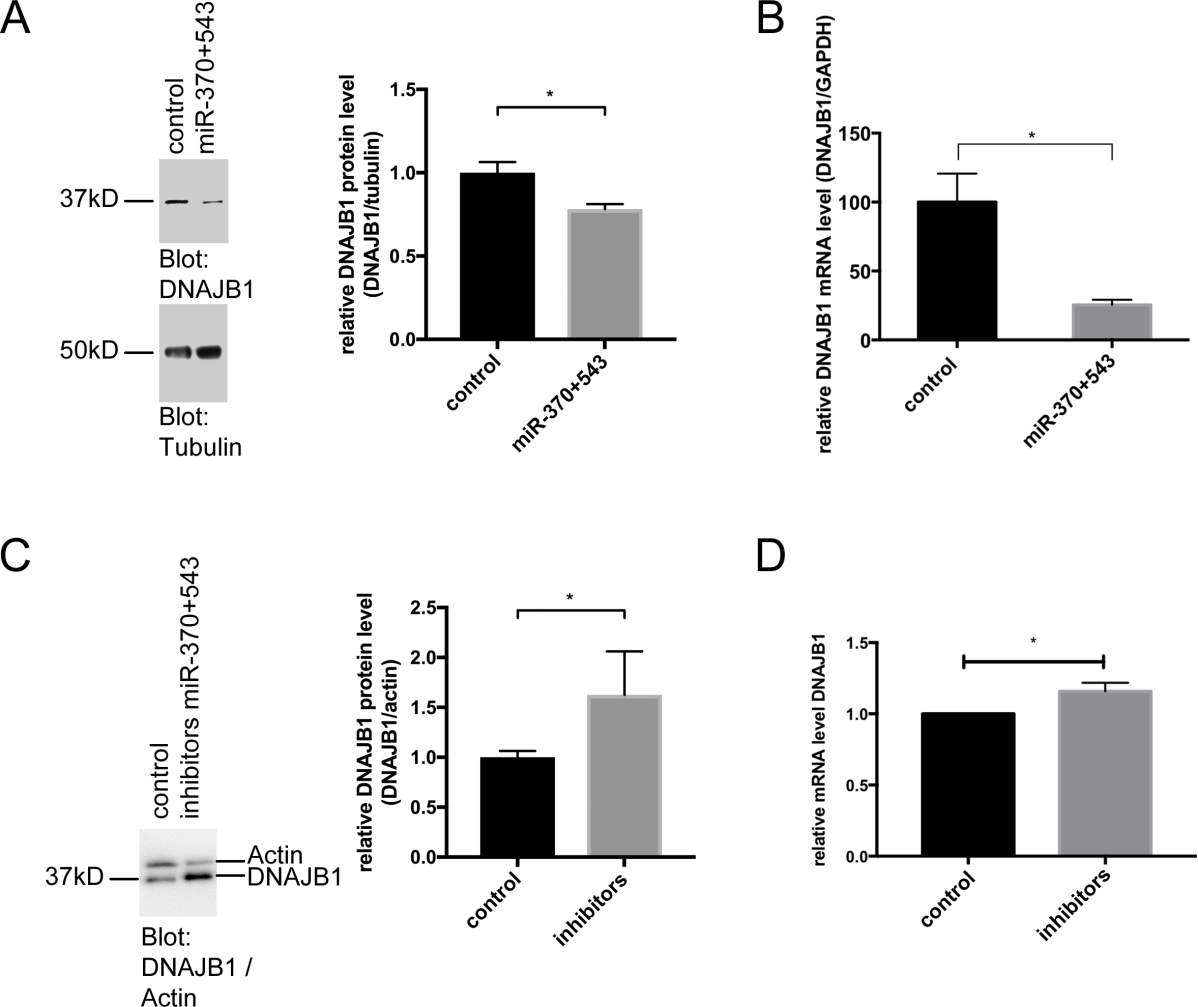
MiR-370 and miR-543 regulate endogenous DNAJB1. (A) A pool of miR-370 and miR-543 mimics was transfected into Hela cells and DNAJB1 protein was detected on a western blot using DNAJB1 specific antibodies. GAPDH was detected as loading control. A representative blot of an n = 4 is shown. p = 0.0439 (B) Realtime-PCR analysis of cells transfected as described in (A). Columns represent mean values +/- SEM of n = 4, p = 0.0047. (C) A pool of miR-370 and miR-543 inhibitors was transfected into Hela cells and DNAJB1 protein was detected on a western blot using DNAJB1 specific antibodies. Actin was detected as loading control. A representative blot of an n = 3 is shown. p = 0.0413 (D) Realtime-PCR analysis of cells transfected as described in (C). Columns represent mean values +/- SEM of n = 7, p = 0.0099.

Next, we tested if overexpression of miR-370 and miR-543 affects expression of endogenous DNAJB1 protein. Therefore, Hela cells were transfected with miRNA mimics (synthetically produced oligonucleotides mimicking endogenously produced miRNAs). Since miRNAs exert their regulatory effects on targets by working in cooperation, a pool of miR-370 and miR-543 mimics was transfected. A significant decrease in DNAJB1 protein levels was observed on western blots after transfection of the mimics pool (Fig 3A). Similarly, the pool of miR-370 and miR-543 also led to a decrease in DNAJB1 mRNA levels as determined by quantitative real-time PCR (Fig 3B). In line, the opposite effect, namely the stabilisation of DNAJB1, was observed after treatment with inhibitors of miR-370 and miR-543, both, on protein (Fig 3C) and on RNA level (Fig 3D), indicating that miR-370 and miR-543 regulate expression of DNAJB1.

### Overexpression of miR-370 and miR-543 and reduced expression of DNAJB1 in SCA3 patient derived cell lines

**Fig 4:**
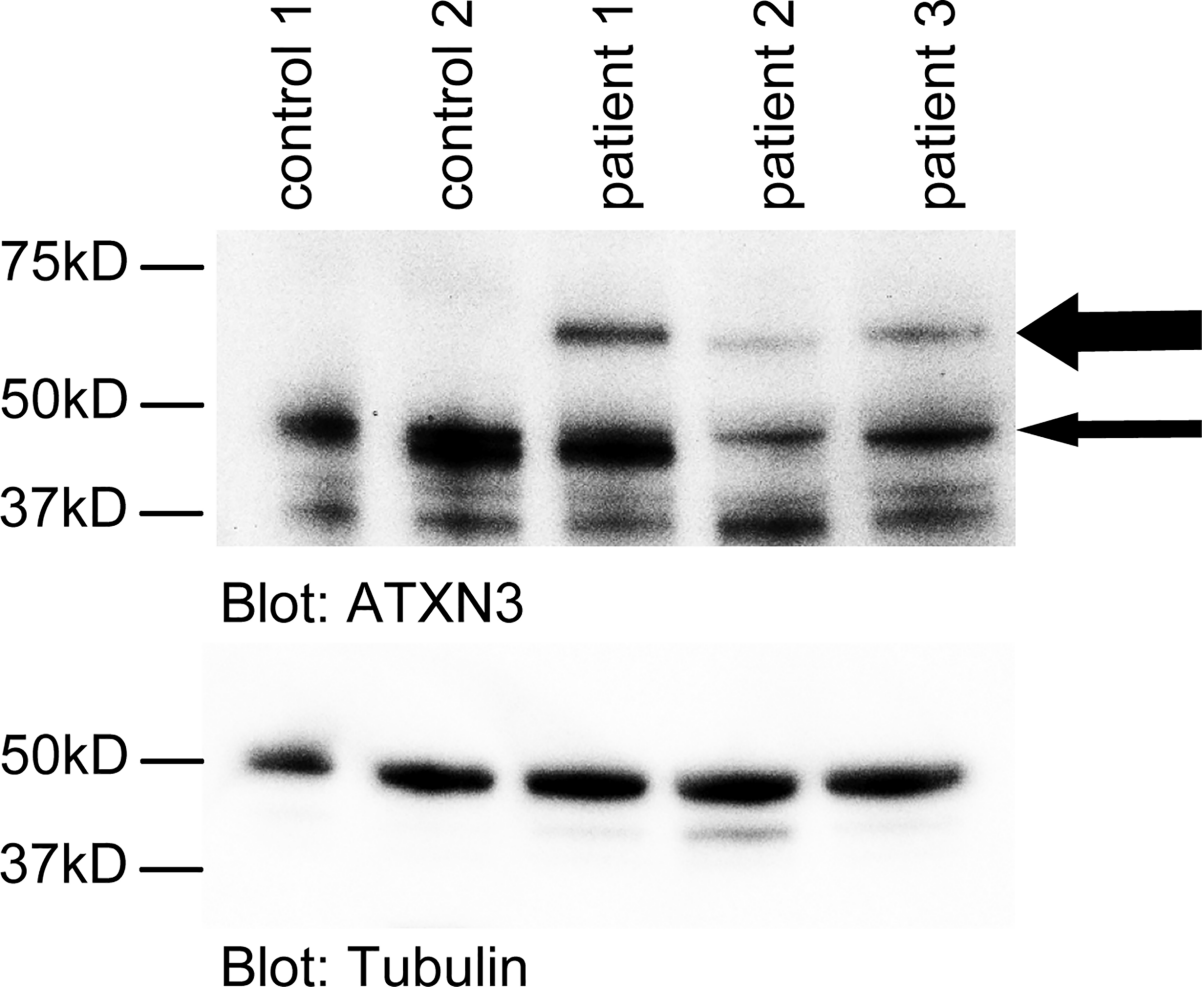
Expanded ATXN3 allele is expressed in differentiated SCA3 lt-NES cells. Western blot analysis of wild type ATXN3 (approx.50 kD, thin arrow) expressed in both the controls and the SCA3 cells, and the expanded, mutant ATXN3 protein (approx.70 kD, thick arrow) in the SCA3 cells.

DNAJB1 has previously shown to colocalize to mutant ATXN3 aggregates [9, 10] and modify polyQ protein toxicity [11]. This suggests that one pathogenic mechanism in SCA3 involves DNAJB1. Thus, we next analyzed expression levels of miRNA-370, 543 and DNAJB1 mRNA in an established long term self-renewing neuroepithelial-like stem (lt-NES) cell-based model of SCA3 derived from human patient-specific induced pluripotent stem cell (iPSC) lines [17]. In this model, excitation of neuronally differentiated SCA3 cells leads to proteolytic cleavage of ATXN3 and formation of ATXN3-containing microaggregates. Three different iPSC-derived SCA3 lt-NES cell lines expressing normal and expanded ATXN3 (Fig 4) and two iPSC lines derived from age- and sex-matched unaffected individuals were used for neuronal differentiation. Total RNA was isolated after 8 weeks of spontaneous differentiation and analyzed either via realtime PCR or with an IlluminaHiSeq2000 with libraries prepared according to the small RNA protocol. Of note, these cells did not undergo excitation and do not contain ATXN3-aggregates and thus represent an early disease-stage prior aggregate-formation.

**Fig 5:**
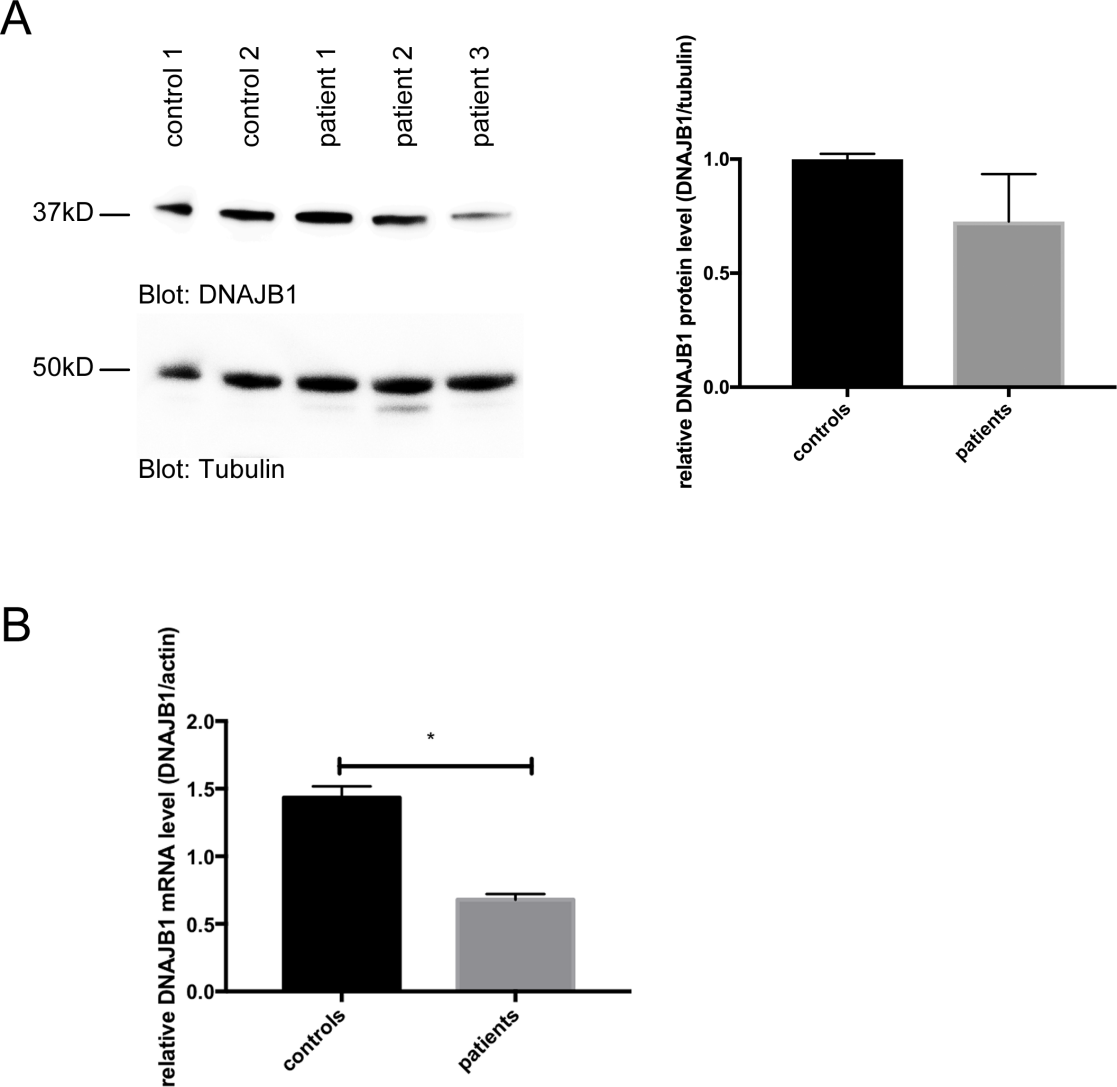
Reduced expression of DNAJB1 in SCA3 patient derived cell lines. (A) DNAJB1 protein levels are slightly reduced in differentiated SCA3 lt-NES cells. DNAJB1 (upper panel) and alpha-tubulin (lower panel) protein levels were determined on western blots. Relative DNAJB1 protein levels were quantified (normalized to alpha-tubulin). Columns represent mean values +/- SD from 2 control neuronal cell lines (controls) and 3 SCA3 neuronal cell lines (patients. (B) DNAJB1 mRNA levels are reduced in the differentiated SCA3 lt-NES cells. DNAJB1 and beta-actin mRNA levels were analyzed by quantitative real-time PCR. Columns represent mean values +/- SD from 2 control neuronal cell lines (controls) and 3 SCA3 neuronal cell lines (patients), p = 0.0008.

Compared to controls, differentiated SCA3 lt-NES cells showed a significant downregulation of the DNAJB1 mRNA levels. In agreement the DNAJB1 protein levels show a trend to be reduced in differentiated SCA3 lt-NES cells compared to controls (Fig 5A and 5B).

The analysis of the miRNA expression profiles revealed that miRNAs miR-370 and miR-543 are upregulated in differentiated SCA3 lt-NES cells (Table 1, S1 Table–S7 Table). Thus, with respect to our observation that miR-370 and miR-543 target DNAJB1 mRNA for degradation, the reduced expression levels of DNAJB1 in differentiated SCA3 lt-NES cells may result from upregulation of miRNAs miR-370 and miR-543.

**Table 1:** miRNAs dysregulated in differentiated SCA3 lt-NES cells with binding sites on the 3’UTR of DNAJB1 mRNA. Expression profiling data of miRNAs miR-370 and miR-543 from 3 SCA3 cell lines and 2 control cell lines.

miRNA name	Fold change in differentiated SCA3 lt-NES cells
hsa-miR-370	45.14309
hsa-miR-543	25.32928

### Reduced DNAJB1 expression levels in a YAC mouse model of SCA3

**Fig 6:**
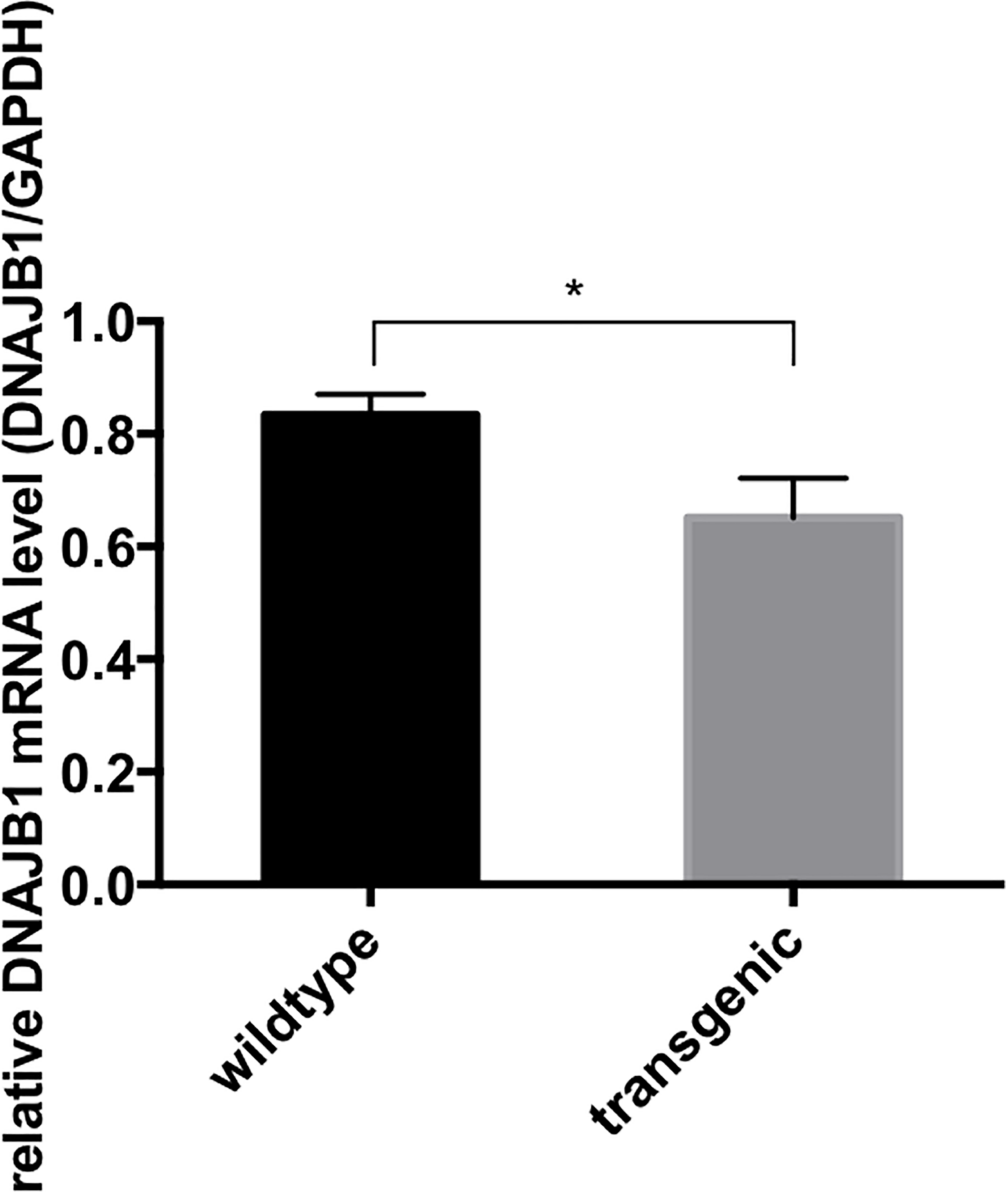
DNAJB1 mRNA levels in the hindbrains of 6 month old transgenic YAC-SCA3 mice and wild type mice. DNAJB1 and GAPDH mRNA levels (as loading control) were checked by SYBR Green realtime PCR. Columns represent values +/- standard deviation. n = 4, P-value = 0.0004.

The DNAJB1 expression levels were also evaluated in a transgenic YAC mouse model of SCA3. This model was previously generated using yeast artificial chromosome (YAC) constructs containing the entire human SCA3 gene locus (250 kb) including the 3’UTR with an expanded (Q84) polyQ tract [19]. SCA3-YAC-84Q mice expressing the human ATXN3 gene with 84 CAG repeats exhibit SCA3 related symptoms such as motor dysfunction, progressive ataxia, and presence of ATXN3 aggregates in neurons [19]. DNAJB1 mRNA levels were analyzed in hindbrains of 6 month-old male control and heterozygous SCA3-YAC-84Q mice. In line with our observation from the human SCA3 cell model, the DNAJB1 mRNA levels were significantly reduced in the SCA3 mice (0.834+/-0.01812 in wildtype versus 0.6514+/-0.03498 in transgenic mice, n = 4) (Fig 6).

## Discussion

Chaperones and the co-chaperone like DNAJB1 play an important role in protein quality control and are known to be involved in the neuropathogenesis of polyQ diseases like SCA3. In this study, we identified two miRNAs, miR-370 and miR-543, that are able to regulate gene expression of DNAJB1 at the post-transcriptional level. In neuronal cultures differentiated from iPSC lines of SCA3 patients, the expression levels of miR-370 and miR-543 were strongly increased while the expression of DNAJB1 was reduced suggesting that both miRNAs act cooperatively in downregulating DNAJB1 expression. Thus, altered expression of miR-370 and miR-543 may contribute to SCA3 pathogenesis by targeting DNAJB1.

So far, little is known about specific miRNAs targeting DNAJB1. The online resource mimirna fails to reveal miRNAs neither positively nor negatively correlated with DNAJB1 [20]. In a recent study differentially expressed miRNAs were identified in human ejaculated spermatozoa potentially regulating expression of heat shock proteins and sperm function [21]. The increased expression levels of DNAJB1 found in sperm are assumed to be negatively correlated with the downregulated expression of miR-449a [21]. In neurons differentiated from iPSC lines of SCA3 patients, miR-449a showed downregulation, however, the DNAJB1 expression levels were not increased but clearly decreased indicating that an inverse functional correlation between miR-449a and DNAJB1 mRNA is unlikely. Another validated miRNA directly targeting DNAJB1 is miR-155-3p, which is highly upregulated in T helper cells during experimental autoimmune encephalomyelitis and controls T helper cell differentiation [22]. In SCA3 patient-specific neuronal cultures differential expression of miR-155-3p was not found. Instead, we identified two miRNAs targeting the 3’UTR of DNAJB1 at specific sites. To our knowledge, this is the first report describing a functional connection between DNAJB1 and miRNAs, miR-370 and miR-543.

Several studies have shown that DNAJB1 is involved in SCA3/polyQ disease pathogenesis. For example in SCA3 patient brain tissue, neurons with intranuclear inclusions show a re-distribution of DNAJB1 protein. DNAJB1 is sequestered from the cytosol to the nuclear inclusions formed by polyQ expanded proteins [9, 10]. Since DNAJB1 is an essential factor for the proteosomal degradation of polyQ proteins via the Hsp70 machinery [23], sequestration of the low-abundant co-chaperone DNAJB1 into nuclear inclusions limits the capability of neurons to degrade neurotoxic polyQ proteins. Reduced expression levels and thus a reduced activity of DNAJB1 may even further promote formation of aggregates. In support of this, it has been shown that overexpression of DNAJB1 restores the cellular degradation capacity [23] and suppresses aggregation of polyQ expanded ATXN3 [24]. However, our findings now indicate that deregulated expression of DNAJB1 and its targeting miRNAs already occurs at early stages of SCA3 disease since they were observed under standard culture conditions where differentiated SCA3 lt-NES cells do not form ATXN3 aggregates [17]. Thus, the mRNA/miRNA changes identified in neurons differentiated from iPSC lines of SCA3 patients are not likely the result of a compromised protein quality control but rather point to early SCA3 disease-related changes in gene expression and gene regulatory networks. In this regard, the aberrant expression of miRNAs targeting DNAJB1 represents an important novel aspect of the disease pathogenesis in SCA3.

In agreement with the reduced expression levels of DNAJB1 in SCA3 patient-specific neuronal cultures, we found significantly reduced DNAJB1 mRNA levels in 6 month old SCA3-YAC-84Q mice. Consistently, a pronounced downregulation of DNAJB1 both at the mRNA and protein level was revealed in 10 to 11 month old transgenic SCA3 mice expressing mutant ATXN3-Q79 [5]. These findings suggest that reduced expression of DNAJB1 constitutes an important part of the pathogenic process in SCA3 disease across species. However, it is currently unknown whether a comparable miRNA-mediated mechanism, as we observed in neurons differentiated from iPSC lines of SCA3 patients, is involved in the reduction of DNAJB1 in transgenic SCA3 mice and awaits future investigation.

In summary our findings suggest for the first time a role for miR-370 and miR-543 and its target *DNAJB1* in the pathogenesis of SCA3. In SCA3 neuronal cultures, expression of miR-370 and miR-543 is increased and expression of DNAJB1 is reduced. Since we observed that miR-370 and miR-543 target DNAJB1, inhibition of these two miRNAs should stabilize the DNAJB1 protein. This in turn should lead to an enhanced capacity of the cellular stress response and reduce polyQ protein toxicity in the disease context. Future studies should aim at pharmacologically inhibiting strategies of miR-370 and miR-543 in SCA3 mouse models to validate whether these two miRNAs represent valid drug targets for a novel therapeutic approach.

## Supporting information

S1 Table List of miRNAs upregulated in iPSC-derived neurons from SCA3 patients in comparison to iPSC-derived neurons from healthy controls. miRNAs have been arranged in descending order of fold change.
(DOCX)

S2 Table List of miRNAs downregulated in iPSC-derived neurons from SCA3 patients in comparison to iPSC-derived neurons from healthy controls. miRNAs have been arranged in descending order of fold change.
(DOCX)

S3 Table Count files for RNAseq data from S1 Table/S2 Table. Count files from control 1.
(TXT)

S4 Table Count files for RNAseq data from S1 Table/S2 Table. Count files from control 2.
(TXT)

S5 Table Count files for RNAseq data from S1 Table/S2 Table. Count files from patient 1.
(TXT)

S6 Table Count files for RNAseq data from S1 Table/S2 Table. Count files from patient 2.
(TXT)

S7 Table Count files for RNAseq data from S1 Table/S2 Table. Count files from patient 3.
(TXT)
