# Supplementary material for: Upregulation of miR-370 and miR-543 is associated with reduced expression of heat shock protein 40 in spinocerebellar ataxia type 3
Source: PLoS One. 2018 Aug 7;13(8):e0201794. doi: 10.1371/journal.pone.0201794 (PMC6080806; doi:10.1371/journal.pone.0201794)
Supplement: S1 Table — miRNAs have been arranged in descending order of fold change. (DOCX) [file pone.0201794.s001.docx]

**S1 Table: List of miRNAs upregulated in differentiated SCA3 lt-NES cells in comparison to controls.** miRNAs have been arranged in descending order of fold change.

| **miRNA name** | **Fold change in differentiated SCA3 lt-NES cells** | **FDR P-value** |
| --- | --- | --- |
| hsa-miR-431-5p | 586.6214 | 1.27E-07 |
| hsa-miR-337-5p | 352.2233 | 3.55E-06 |
| hsa-miR-770-5p | 191.9895 | 7.60E-07 |
| hsa-miR-376a-3p | 182.4585 | 1.78E-06 |
| hsa-miR-539-5p | 134.6055 | 7.88E-05 |
| hsa-miR-432-5p | 85.25497 | 6.68E-07 |
| hsa-miR-432-3p | 84.53678 | 2.02E-03 |
| hsa-miR-541-5p | 83.10655 | 3.44E-02 |
| hsa-miR-127-3p | 80.14982 | 6.16E-13 |
| hsa-miR-541-3p | 78.95533 | 4.87E-04 |
| hsa-miR-337-3p | 74.32188 | 3.41E-08 |
| hsa-miR-136-5p | 66.85646 | 2.62E-10 |
| hsa-miR-380-5p | 66.44027 | 2.40E-03 |
| hsa-miR-299-5p | 66.33228 | 3.67E-09 |
| hsa-miR-369-5p | 65.50373 | 1.28E-09 |
| hsa-miR-127-5p | 65.33154 | 1.21E-11 |
| hsa-miR-409-3p | 62.79913 | 2.31E-06 |
| hsa-miR-154-5p | 61.49923 | 1.98E-09 |
| hsa-miR-136-3p | 58.97185 | 6.01E-12 |
| hsa-miR-485-3p | 54.48456 | 7.84E-07 |
| hsa-miR-654-3p | 54.26493 | 1.61E-08 |
| hsa-miR-380-3p | 52.58121 | 1.28E-09 |
| hsa-miR-495-3p | 51.78691 | 1.98E-09 |
| hsa-miR-379-3p | 51.31836 | 8.21E-09 |
| hsa-miR-409-5p | 50.65953 | 7.99E-04 |
| hsa-miR-433 | 50.51679 | 9.45E-06 |
| hsa-miR-758-3p | 49.5244 | 8.13E-09 |
| hsa-miR-134 | 48.39775 | 2.92E-07 |
| hsa-miR-369-3p | 48.19559 | 4.15E-10 |
| hsa-miR-379-5p | 48.05916 | 2.62E-10 |
| hsa-miR-377-5p | 47.36093 | 6.32E-07 |
| hsa-miR-382-3p | 47.25587 | 3.38E-09 |
| hsa-miR-323b-3p | 46.76528 | 3.01E-02 |
| hsa-miR-382-5p | 45.61819 | 3.20E-07 |
| hsa-miR-494 | 45.15569 | 7.63E-07 |
| hsa-miR-370 | 45.14309 | 6.47E-06 |
| hsa-miR-323a-3p | 43.38047 | 5.32E-06 |
| hsa-miR-1197 | 42.06295 | 6.41E-06 |
| hsa-miR-668 | 41.84241 | 1.86E-03 |
| hsa-miR-654-5p | 41.30734 | 7.63E-07 |
| hsa-miR-544a | 40.98868 | 2.50E-02 |
| hsa-miR-431-3p | 40.23896 | 1.45E-08 |
| hsa-miR-376b-5p | 39.76311 | 3.05E-02 |
| hsa-miR-411-5p | 39.52897 | 2.62E-10 |
| hsa-miR-1185-5p | 39.2931 | 1.09E-09 |
| hsa-miR-381-5p | 39.1471 | 3.45E-02 |
| hsa-miR-889 | 38.68175 | 2.62E-10 |
| hsa-miR-487b | 38.63271 | 1.28E-09 |
| hsa-miR-485-5p | 38.617 | 6.96E-07 |
| hsa-miR-411-3p | 38.05624 | 3.38E-09 |
| hsa-miR-410 | 37.85289 | 1.00E-05 |
| hsa-miR-376c-3p | 37.81475 | 1.05E-09 |
| hsa-miR-1185-1-3p | 37.36638 | 3.41E-08 |
| hsa-miR-656 | 36.59561 | 9.33E-09 |
| hsa-miR-376a-5p | 36.54073 | 2.30E-04 |
| hsa-miR-1185-2-3p | 36.33916 | 6.35E-04 |
| hsa-miR-381-3p | 35.94446 | 3.26E-10 |
| hsa-miR-329 | 35.7301 | 7.63E-08 |
| hsa-miR-377-3p | 35.65163 | 3.41E-08 |
| hsa-miR-376b-3p | 33.61442 | 1.72E-07 |
| hsa-miR-299-3p | 33.5104 | 2.86E-09 |
| hsa-miR-539-3p | 32.90574 | 2.01E-09 |
| hsa-miR-487a | 29.85199 | 6.70E-09 |
| hsa-miR-543 | 25.32928 | 4.73E-04 |
| hsa-miR-496 | 25.30281 | 5.99E-05 |
| hsa-miR-758-5p | 21.16692 | 9.54E-03 |
| hsa-miR-493-5p | 20.73649 | 3.19E-07 |
| hsa-miR-493-3p | 20.04763 | 4.52E-07 |
| hsa-miR-655 | 19.30396 | 1.86E-05 |
| hsa-miR-2113 | 6.406751 | 2.18E-03 |
| hsa-miR-490-3p | 4.952 | 2.08E-03 |
