# Supplementary material for: Upregulation of miR-370 and miR-543 is associated with reduced expression of heat shock protein 40 in spinocerebellar ataxia type 3
Source: PLoS One. 2018 Aug 7;13(8):e0201794. doi: 10.1371/journal.pone.0201794 (PMC6080806; doi:10.1371/journal.pone.0201794)
Supplement: S2 Table — miRNAs have been arranged in descending order of fold change. (DOCX) [file pone.0201794.s002.docx]

**S2 Table: List of miRNAs downregulated in differentiated SCA3 lt-NES cells in comparison to controls.** miRNAs have been arranged in descending order of fold change.

| **miRNA name** | **Fold change in differentiated SCA3 lt-NES cells** | **FDR P-value** |
| --- | --- | --- |
| hsa-miR-449a | -108.2534889 | 1.78E-03 |
| hsa-miR-449c-5p | -93.32553078 | 9.54E-03 |
| hsa-miR-449b-5p | -80.34681096 | 1.78E-03 |
| hsa-miR-199a-5p | -66.25275354 | 3.01E-02 |
| hsa-miR-4490 | -65.61140287 | 3.85E-03 |
| hsa-miR-199a-3p | -61.29128195 | 3.47E-02 |
| hsa-miR-199b-3p | -61.29128195 | 3.47E-02 |
| hsa-miR-199b-5p | -48.63167017 | 4.46E-02 |
| hsa-miR-2114-5p | -19.71020476 | 2.19E-03 |
| hsa-miR-143-3p | -17.50185064 | 5.00E-02 |
| hsa-miR-193a-3p | -13.50312411 | 2.02E-02 |
| hsa-miR-145-5p | -12.31148516 | 4.63E-02 |
| hsa-miR-2114-3p | -12.08693102 | 1.17E-03 |
| hsa-miR-1266 | -4.295350381 | 3.81E-02 |
| hsa-miR-1269a | -4.090803361 | 8.91E-03 |
| hsa-miR-184 | -3.930027022 | 2.27E-02 |
| hsa-miR-190b | -3.869430519 | 2.55E-02 |
| hsa-miR-27b-5p | -3.524804277 | 2.55E-02 |
| hsa-miR-23b-3p | -3.287689506 | 3.47E-02 |
